# Composite surface roughness and color change following airflow usage

**DOI:** 10.1186/s12903-021-01745-3

**Published:** 2021-08-14

**Authors:** Azam Valian, Zahra Jaber Ansari, Mohammad Moien Rezaie, Roja Askian

**Affiliations:** Department of Restorative Dentistry, School of Dentistry, School of Dentistry, Shahid Beheshti University of Medical Sciences, Daneshjoo Blvd, Evin, Shahid Chamran Highway, Tehran, 1983963113 Iran

**Keywords:** Airflow, Composite resins, Polishing, Surface roughness

## Abstract

**Background:**

Esthetic dental restorations have gained increasing popularity. The surface of restorations should be smooth enough to achieve maximum esthetics and prevent the adhesion of microorganisms and food particles. This study aimed to assess the surface roughness and color change of composite specimens following airflow usage.

**Methods:**

In this in vitro, experimental study, 30 Tokuyama composite discs were fabricated and randomly divided into three groups (n = 10) for the use of airflow with calcium carbonate/bicarbonate powder and conventional polishing with FlexiDisc. The surface roughness of the specimens was measured by profilometry while the color change was assessed by measuring the L*, a* and b* color parameters using spectrophotometry before polishing (T1). The composite specimens were then polished for stain removal, and their surface roughness as well as color parameters were remeasured after polishing (T2). Paired t-test and Tukey’s test were applied for within-group and between-group comparisons.

**Results:**

Significant differences were noted in roughness average (Ra) between airflow with calcium carbonate (0.251 ± 0.014 μm) and airflow with sodium bicarbonate (0.421 ± 0.208 μm), and between airflow with sodium bicarbonate and FlexiDisc (0.207 ± 0.076 μm) groups after polishing (*P* < 0.05). Regarding the correlation of change in surface roughness and color parameters at T1 and T2, an inverse correlation was noted between the change in surface roughness and all color parameters except for L*. In other words, reduction in surface roughness decreased the a* and b* color parameters.

**Conclusions:**

Within the limitations of this study, the results showed that the airflow device used in this study had no significant difference with conventional polishing in terms of reduction in surface roughness and staining. Considering the cost and maintenance of the airflow device, it is not suggested as a suitable alternative to the conventional polishing procedures.

*Trial Registration Number*: This study does not involve human subjects.

## Background

Demand for a beautiful smile has greatly increased in the past decades [1]. Tooth color is a fundamental factor in achieving an attractive smile [2–4]. A poor-quality restoration can cause several complications [5]. Development of secondary caries and discoloration of restoration can lead to failure and eventual tooth loss [6].

Staining is an important factor that can compromise the esthetic appearance of teeth and restorations [7]. Staining can be intrinsic or extrinsic. Extrinsic staining occurs following the deposition of metallic or non-metallic stains on the tooth surface [8, 9]. Consumption of iron drops can lead to deposition of iron stains on the tooth surface while non-metallic staining may occur due to the effects of chromogenic bacteria, tobacco smoke, consumption of tea, coffee or cola, intake of some medications, use of mouth rinses, and also some restorative materials, and greatly depends on the nutritional habits of individuals [10, 11].

Several methods have been suggested for stain removal, which can be adopted depending on the depth and severity of staining [12, 13]. Airflow or air-polishing devices are among the novel techniques suggested for elimination of stains, and surface polishing [14–17]. Their mechanism of action is based on abrasion by micron-scale cubic or spherical particles. The manufacturers claim that these devices can provide a smoother surface compared with the conventional methods, if used according to the manufacturers’ instructions [16]. A more commonly employed method is to use a combination of diamond paste (FGM, Brazil) with extra-fine 6-µm diamond particles and buff disc [17]. Also, FlexiDisc dental finishing and polishing discs (Cosmedent, Germany) are used for composite polishing with aluminum oxide particles with different grits [18].

Profilometry, microradiography, atomic force microscopy, and scanning electron microscopy are commonly used for assessment of surface roughness. Profilometry uses a contact probe to measure the surface roughness of a certain area in nanometers [19, 20].

The three-dimensional CIE L*a*b* color space is conventionally used for assessment of change in tooth color [21, 22]. Spectrophotometry is a technique for assessment of electromagnetic spectra. It measures the light absorption by specimens to determine the L*, a* and b* color parameters. The L* coordinate indicates lightness, ranging from 0 (black) to 100 (white). The positive a* values indicate redness and the negative values indicate greenness. The positive b* parameters indicate yellowness and the negative values indicate blueness. The color difference (ΔE) between the two objects is calculated in the CIE L*a*b* color system.

Considering the increasing demand for esthetic dental restorations, finding a method to enhance the durability and improve the esthetic appearance of restorations by minimizing their discoloration is a priority. Thus, this study assessed the composite surface roughness and color change following airflow usage. The possible correlation of surface roughness with staining after polishing was also evaluated. The null hypothesis was that there would be no difference in surface roughness of composite resin subjected to airflow or the conventional polishing.

## Methods

A total of 30 composite discs were evaluated in three groups including 20 specimens in airflow groups with calcium carbonate spherical particles (0.251 ± 0.014 μm) and sodium bicarbonate cubic particles (0.421 ± 0.208 μm) and 10 specimens in the control group for conventional polishing with FlexiDisc (0.207 ± 0.076 μm). Composite discs (BW shade Estellite Sigma Quick; Tokoyama Dental, Tokoyama, Japan) were fabricated measuring 8 × 2 mm.

In order to standardize the range of movement of the profilometer probe for all specimens at the two assessment time points, an area with 4 mm diameter was outlined at the center of each specimen using a 010 long, flat-end cylindrical bur (Tiz Kavan, Iran). A line was drawn vertical to this area to standardize the movement of profilometer probe on the surface of all specimens (the depth of lines was half the diameter of the bur). The surface of specimens was then polished with a yellow mullet (Toboom, China) and a handpiece operating at 600 rpm for 30 s under water coolant [23]. All procedures were performed by the same operator.

Next, the primary surface roughness of specimens was measured, and baseline color assessment was performed. The primary L*, a* and b* parameters and surface roughness were all recorded. Surface roughness was measured by profilometry (Dektak XT stylus profiler (Bruker, USA). Next, half of the specimens underwent artificial staining in tea solution, which was prepared by adding 9 g of loose black tea (Ahmad) to 450 mL of boiling distilled water for 5 min [19]. Each specimen was immersed in tea solution for 7 days at room temperature; during this period, the tea solution was replaced with fresh tea solution on a daily basis. After 7 days, the specimens were removed from the solution and rinsed with distilled water. Next, different polishing techniques (airflow, diamond paste, and FlexiDisc) were applied for stain removal.

The specimens underwent airflow polishing (Prophy-Mate Neo, NSK, Japan) for 20 s at 3 mm distance from the surface at 10° angle [8] under water and air spray. The distance from the surface was standardized by using a graded rod next to the nozzle of the device.

The composite specimens in the control group underwent polishing with FlexiDisc (Cosemdent, Germany). The blue (medium), yellow (fine) and pink (extra fine) colors of the discs were used in an orderly manner. Each disc was used for 10 s with a handpiece operating at 600 rpm under water coolant. The specimens were rinsed with water for 5 s after using each disc [23].

The specimens were then sent again to a laboratory for assessment of surface roughness and color measurement (T2). The following formula was used to calculate the ∆E:

$$\Delta {\text{E}}=[(\Delta {\text{a}}^*)^2+(\Delta {\text{b}}^*)^2+(\Delta {\text{L}}^*)^2]^0.$$

### Statistical analysis

Data were analyzed using SPSS version 23. Normal distribution of data was evaluated using the Kolmogorov-Smirnov test. The mean Surface roughness and ΔE parameters were calculated and reported for each group. Paired t-test was used for within-group comparisons between different time points. The correlation test was applied to assess the correlation of surface roughness and color change. *P* < 0.05 was considered statistically significant.

## Results

In this study, 30 specimens in three groups (n = 10) underwent airflow (with two powders) and FlexiDisc polishing methods. Data were collected using a profilometer and a spectrophotometer at baseline (T1) and also after polishing (T2).

### Assessment of surface roughness in the study groups at different time points

Table 1 shows the surface roughness average (Ra) of the study groups at different time points. As shown, within-group comparison of surface roughness parameter revealed its significant change over time in the calcium carbonate (*P* = 0.01) and FlexiDisc (*P* = 0.01) groups. Comparison of surface roughness of composite specimens at the two time points by Tukey’s test revealed a significant difference between calcium carbonate and sodium bicarbonate (*P* = 0.02), and also between sodium bicarbonate and FlexiDisc (*P* = 0.01) groups after polishing.

**Table 1:** Within-group comparison of surface roughness parameter in the study groups at different time points

Group	Time		
	Ra (µm) (T1)	Ra (µm) (T2)	(*P* value)
Calcium carbonate	0.334 ± 0.071	0.251 ± 0.014	0.01
Sodium bicarbonate	0.393 ± 0.055	0.421 ± 0.208	0.471
FlexiDisc	0.345 ± 0.029	0.207 ± 0.076	0.019

T1, before polishing; T2, after polishing; Ra, Roughness average

**Fig. 1:**
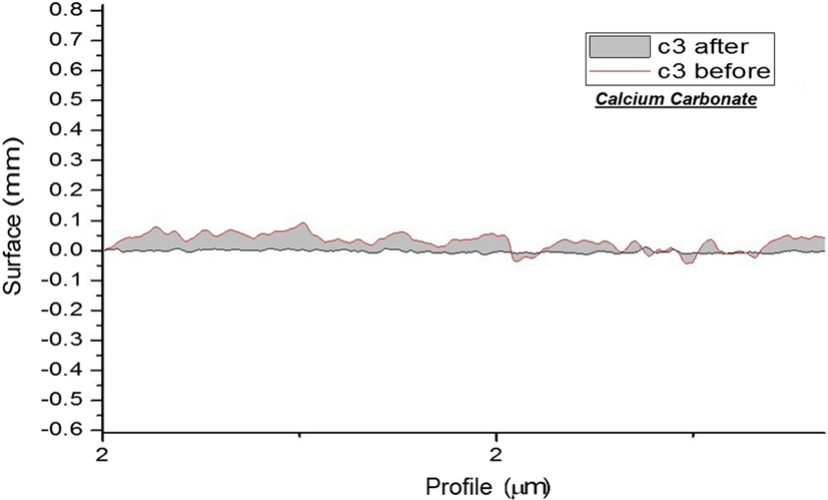
Topographic profile of composite before and after polishing by airflow (calcium carbonate)

**Fig. 2:**
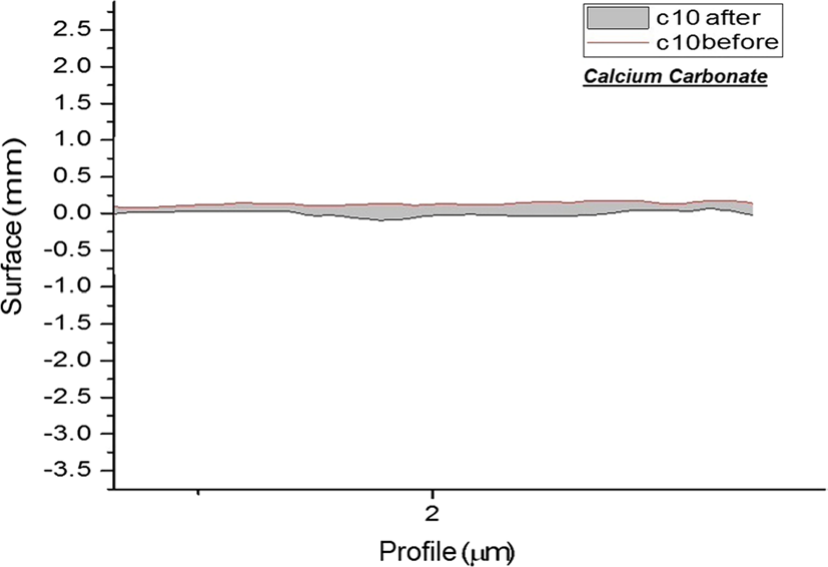
Topographic profile of composite before and after polishing by airflow (calcium carbonate)

**Fig. 3:**
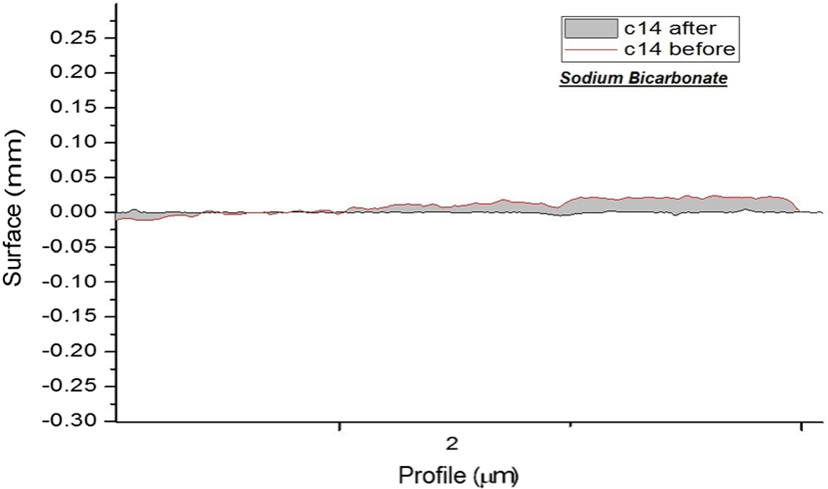
Topographic profile of composite before and after polishing by airflow (sodium bicarbonate)

**Fig. 4:**
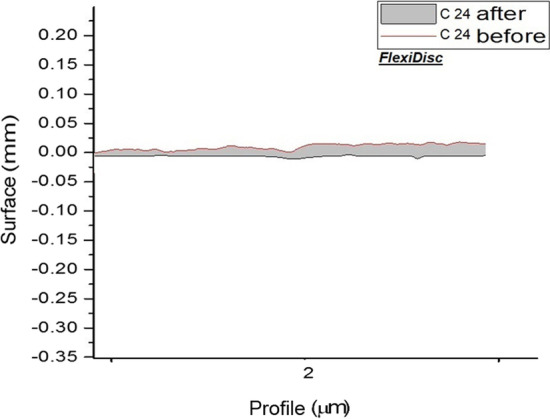
Topographic profile of composite before and after polishing by FlexiDisc

And surface roughness of each group are shown separately in Figs. 1, 2, 3 and 4.

### Assessment of ∆E of the groups at different time points

Table 2 presents the ∆E of the study groups at different time points. Comparison of ∆E at the two time points among the polishing groups by the Tukey’s test revealed no significant difference (*P* = 0.713).

**Table 2:** Comparison of ∆E of the study groups at different time points

Group	Time		
	∆E1	∆E2	*P* value (∆E1 − ∆E2)
Airflow with calcium carbonate	5.33 ± 2.37	4.13 ± 3.63	0.021
Airflow with sodium bicarbonate	5.08 ± 3.85	4.57 ± 2.77	0.861
FlexiDisc	5.21 ± 2.72	4.19 ± 2.81	0.618

∆E: Color difference between the two objects calculated in the CIE L*a*b* color system

### Assessment of the correlation of surface roughness with ∆E before and after polishing

The correlation of change in surface roughness with color parameters was evaluated before (T1) and after (T2) polishing. The results showed that except for the L* parameter, other color parameters had an inverse correlation with the change in surface roughness, and decreased by an increase in surface roughness difference (smoothing of the surface). Surface roughness had significant correlations with ∆E in calcium carbonate group, and the L* and a* parameters in sodium bicarbonate (*P* = 0.02 and *P* = 0.009, respectively) and also in FlexiDisc (*P* = 0.04, Table 3) groups.

**Table 3:** Correlation of surface roughness change with color parameters

Correlation of surface roughness with group	L*	a*	b*	∆E
Airflow with calcium carbonate	0.08	− 0.694	− 0.18	− 0.534
Airflow with sodium bicarbonate	0.335	− 0.543	− 0.415	− 0.282
FlexiDisc	0.351	− 0.743	− 0.412	− 0.649

L* for perceptual lightness, and a* and b* for the four unique colors of human vision: red, green, blue, and yellow

## Discussion

**Fig. 5:**
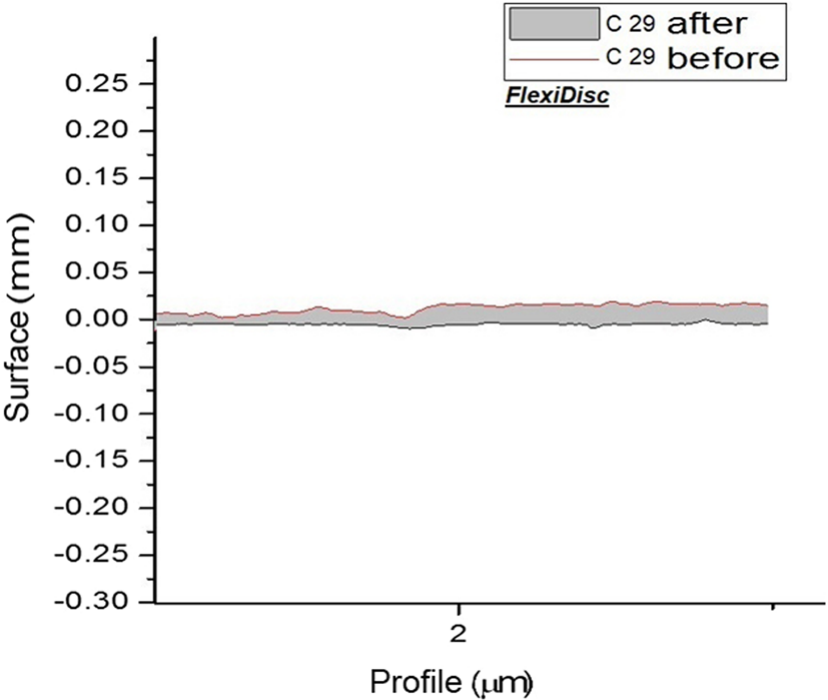
Topographic profile of composite before and after polishing by FlexiDisc

Esthetic dental restorations have gained increasing popularity in the recent years. Although such treatments can meet patient satisfaction in short-term, the important point is to ensure their long-term durability and quality [11] (Fig. 5).

Poorly finished and polished restoration surfaces can enhance the accumulation of bacterial plaque and calculus and lead to subsequent complications [12]. Airflow or air-polishing systems have recently gained popularity for mechanical stain removal. These systems spray compressed air with some powders on the surface to remove the stains [8]. This study assessed the effect of Prophy-Mate Neo airflow device on surface roughness and its efficacy for stain removal from the composite surface. Calcium carbonate and sodium bicarbonate powders can be used with this device. Calcium carbonate particles are spherical and minimize surface traumatization by rolling on the surface. Sodium bicarbonate powder, which is currently less commonly used, has cubic particles. Controversy exists regarding the positioning of cubic particles on the surface [24, 25].

Two other commonly used polishing methods in the clinical setting were also assessed in this study namely the use of diamond paste and buff, and FlexiDisc composite finishing and polishing discs. According to Yurdaguven et al. [11] the Diamond Excel paste applied with a buff disc can yield a lower surface roughness in the enamel than other polishing pastes. Also, Camboni and Donnet [10] found no significant difference in enamel surface roughness following the use of airflow device and diamond paste. Berger et al. [23] showed that FlexiDisc finishing and polishing discs yielded a lower surface roughness than other composite polishing methods, and therefore, were used in this study.

Assessment of surface roughness in the present study revealed a significant change after using airflow with calcium carbonate, compared with the baseline surface roughness of composite specimens (*P* = 0.019). This finding indicated the more successful performance of calcium carbonate in reduction of surface roughness, which was in agreement with the results of Graumann et al. [26]. Superior performance of calcium carbonate powder may be due to the spherical shape of its particles, which can more easily roll on the surface than cubic sodium bicarbonate particles. Also, spherical particles have smaller contact area with the surface and consequently less friction while both spherical and cubic particles of the two powders are of the same size.

Comparison of different composite polishing techniques namely airflow polishing with calcium carbonate, airflow polishing with sodium bicarbonate, and FlexiDisc regarding the resultant surface roughness revealed that airflow with sodium bicarbonate had significant differences with airflow with calcium carbonate and FlexiDisc. This finding could be attributed to the poor performance of sodium bicarbonate powder. Similarly, Barnes et al. [27] discussed that sodium bicarbonate powder yielded higher surface roughness than calcium carbonate and glycine powders. Sulieman et al. [14] in a systematic review discussed that the acceptable surface roughness to obtain a durable and shiny surface was 0.2 μm. In this study, the surface roughness of composite specimens subjected to calcium carbonate (0.251 μm) and FlexiDisc polishing kit (0.207 μm) was close to this value. The difference in surface roughness of enamel (0.381 μm for specimens subjected to calcium carbonate powder and 0.447 μm for specimens subjected to sodium bicarbonate) and composite (0.421 μm in sodium bicarbonate group) can question the efficacy and application of this particular device, compared with other devices and polishing systems, after taking into account its cost and availability. Also, a significant difference in surface roughness was noted in composite specimens subjected to FlexiDisc polishing system compared with baseline (*P* = 0.01). Similarly, Berger et al. [23] reported that application of FlexiDisc and Sof-Lex discs for polishing significantly changed the surface roughness of specimens compared with baseline. However, the mean surface roughness value of composite in our study (0.207 μm in FlexiDisc group) was higher than the value reported by Berger et al. [23] (0.116 μm), which can be due to the different composite types used in the two studies.

Color change of specimens at different time points was determined by calculation of ∆E. The ∆E > 2.7 is often considered as clinically detectable color change [22]. Accordingly, at T1 and T2 following the use of airflow, 97 % of specimens showed discoloration, which indicates successful performance of airflow with both calcium carbonate and sodium bicarbonate powders for stain removal. This finding was in agreement with the results of Graumann et al. [26] regarding the successful results of air-polishing for stain removal.

The relationship of change in surface roughness and color change of specimens was also evaluated in this study. In general, a correlation was noted between the smoothness of the surface and lighter color (L*), which is probably due to the more regular reflection of light due to lower surface porosities. Joiner et al. [25] also reported a correlation between surface smoothness and increase in L* parameter. However, this correlation was only significant for the L* and ∆E of composite specimens subjected to calcium carbonate (*P* = 0.03).

Evaluation of surface roughness and color parameters in the polishing groups using airflow device (Prophy-Mate Neo) revealed successful performance of this device in combination with calcium carbonate powder for composite specimens. Similarly, Graumann et al. [26] confirmed successful performance of calcium carbonate powder for this purpose. However, the cost, maintenance and safety of this device should also be taken into account, which was out of the scope of this study. The conventional clinical methods can also be used with optimal clinical efficacy.

## Conclusions

The Prophy-Mate Neo air-polishing device used with calcium carbonate powder in this study was successful in terms of the resultant surface roughness of composite specimens after polishing compared with baseline. Regarding color change, this device decreased the staining of composite specimens; however, considering its performance with regard to surface roughness, it cannot be recommended as the gold standard for this purpose (yielding a surface roughness higher than 0.2 μm).

Considering the absence of a significant difference between the performance of airflow device and the conventional polishing technique, as well as its cost and maintenance, this device with the studied powders cannot be recommended as an alternative to the currently applied conventional polishing techniques.

## Data Availability

All materials described in this manuscript including all relevant raw data, will be freely available to any scientist wishing to use them for non-commercial purposes, without breaching participant confidentiality. Requests for access to these data should be made to Roja Askian.

## Abbreviations

T1: Before polishing
T2: After polishing
Ra: Roughness average
L: Lightness
a: Redness
b: Yellowness
